# Simplified Insertion of Transgenes Onto Balancer Chromosomes via Recombinase-Mediated Cassette Exchange

**DOI:** 10.1534/g3.112.002097

**Published:** 2012-05-01

**Authors:** Florence F. Sun, Justine E. Johnson, Martin P. Zeidler, Jack R. Bateman

**Affiliations:** * Biology Department, Bowdoin College, Brunswick, Maine 04011; † MRC Centre for Developmental and Biomedical Genetics, University of Sheffield, Sheffield S10 2TN, United Kingdom

**Keywords:** RMCE, targeted transgenesis, phiC31, *Drosophila*, balancer

## Abstract

Balancer chromosomes are critical tools for *Drosophila* genetics. Many useful transgenes are inserted onto balancers using a random and inefficient process. Here we describe balancer chromosomes that can be directly targeted with transgenes of interest via recombinase-mediated cassette exchange (RMCE).

In *Drosophila*, balancer chromosomes bearing multiple inversions are routinely used in genetic manipulations and in the maintenance of sterile or lethal mutations as balanced heterozygotes. Balancer chromosomes typically carry dominant markers, the most common of which affect adult structures only. However, using transgenic approaches, many new markers and functions have been assigned to balancers in efforts to improve their utility. For example, transgenic insertions have been created to facilitate the identification of balanced progeny at different stages of development, including balancers that carry histological or fluorescent markers driven by embryonic enhancers (including so-called “blue” and “green” balancers) (Casso *et al.* 2000; Halfon *et al.* 2002; Le *et al.* 2006; Panzer *et al.* 1993; Rudolph *et al.* 1999). Balancers carrying transgenic insertions of GAL80, a repressor of the UAS/GAL4 system, function similarly in cross schemes involving transgenes driven by UAS (Vef *et al.* 2006). More recently, the cloning of the gene responsible for *Tubby^1^*, a convenient marker that is visible during larval development and is carried on the third chromosome balancer *TM6B*, has led to the creation of *Tubby^1^* transgenes inserted onto X and second chromosome balancers (Guan *et al.* 2006; Lattao *et al.* 2011; Pina and Pignoni 2012). In addition to novel markers for the identification of balanced progeny, others have created transgenic insertions on balancer chromosomes for the convenient delivery of key enzymes into genetic schemes; these include transposases for P (Lindsley and Zimm 1992) and Minos (Metaxakis *et al.* 2005) transposon systems, and Cre (Siegal and Hartl 1996) and FLP (Chou and Perrimon 1992) recombinases. Furthermore, autosomal balancers have been engineered to carry the cell death promoter *hid* in an effort to simplify fly sorting during gene replacement by homologous recombination (Huang *et al.* 2008). Thus, a pattern exists in which the development of new genetic technologies consistently leads researchers to target new transgenes to *Drosophila* balancer chromosomes.

For each of the examples listed above, transgenic insertions were incorporated onto balancers using *P*-element–mediated transgenesis (Rubin and Spradling 1982). *P*-element insertion occurs in an untargeted manner; thus, obtaining transgenes on a balancer requires one to create many independent insertions, and then to screen for those that happened to insert onto the balancer chromosome. This approach typically involves several generations of crosses and requires many lines to be discarded, representing wasted effort and resources. Therefore, we sought to create balancer chromosomes that could be directly targeted with transgenes of interest using phiC31-mediated RMCE (Bateman *et al.* 2006). This approach makes use of a “target cassette,” which consists of a dominant marker gene flanked by attP recognition sites for phiC31 integrase, that is first integrated into the genome. Once the target is established, a “donor cassette” carrying a transgene of interest flanked by phiC31 attB sites can be directly incorporated at the precise genomic position of the target cassette. As described below, our strategy was to use traditional *P*-element–mediated transgenesis to incorporate dominantly marked RMCE target cassettes onto balancer chromosomes. Once established, these targets can be used to directly incorporate transgenes of interest onto balancer chromosomes.

We previously created a *P* element that carries an RMCE target cassette consisting of a *mini-white* gene flanked by phiC31 attP sites (Bateman and Wu 2008). Using a Δ2-3 transposase source, we remobilized existing insertions of this *P* element and, via three different cross schemes, screened for new insertions onto the X-chromosome balancer *FM7h* (Heitzler 1997), the second chromosome balancer *CyO*, and the third chromosome balancer *TM3* (see supporting information, File S1 for details of remobilization). For each balancer, we isolated three independent insertions and used inverse PCR and sequence analysis to map the precise genomic positions of the *P* elements (Table 1). The majority of these insertions mapped to euchromatic regions in or near broadly expressed genes and within chromatin environments expected to facilitate gene expression (Filion *et al.* 2010; Graveley *et al.* 2011; Kharchenko *et al.* 2011) (Table 1, Table S1). One exception, the insertion in line *CyO^J01^*, was mapped to a *Doc* element that we did not precisely locate.

**Table 1:** Balancer chromosomes carrying RMCE target cassettes

Balancer Line	Insert Cytology	Insert Position	Insert Strand	Nearest Gene	Relative Position of Insertion
*FM7h^FS2^*	10B6	X:11266889	Top	*Dlg1*	Genic
*FM7h^FS4^*	18A3-4	X:19047768	Top	*RhoGAP18B*	Genic
*FM7h^FS5^*	6F3	X:6969300	Top	*Sxl*	Intergenic
*CyO^J01^*	ND	ND	ND	*Doc Element*	(Repeat sequence)
*CyO^J04^*	37B8	2L:18987255	Top	*CG10641*	Genic
*CyO^J08^*	37F2	2L:19572635	Bottom	*Spi*	Genic
*TM3^FS10^*	79A2	3L:21872640	Top	*Mub*	Genic
*TM3^FS11^*	85A5	3R:4502640	Top	*CG8043*	Genic
*TM3^FS18^*	100D1	3R:27550495	Bottom	*ttk*	Genic

Insertions were mapped by comparing sequences of inverse PCR products to release 5.3 of the *Drosophila melanogaster* genome sequence. The insertion in the *CyO^J01^* line was found in a *Doc* element and was not precisely mapped (ND, not determined). See Table S1 for further information on genome annotations near each insertion.

To assess the potential utility of these lines, we first confirmed that RMCE was supported at appreciable levels using at least one representative target-bearing line for each of the three balancer chromosomes, namely *FM7h^FS5^*, *CyO^J01^*, *CyO^J08^*, and *TM3^FS18^*. In the presence of a genomic source of the phiC31 integrase (Bischof *et al.* 2007), we injected donor constructs carrying attB sites flanking either an intronless *yellow* gene or a fluorescent marker driven by the eye-specific enhancer *GMR* (Moses and Rubin 1991) (Figure 1 and Table 2). Although we found experimental variation in transformation efficiencies, the lines that we tested supported RMCE at rates up to 41%, consistent with our rates of transgenesis for other genomic targets using this method and our current injection apparatus (data not shown; Bateman and Wu 2008).

**Figure 1:**
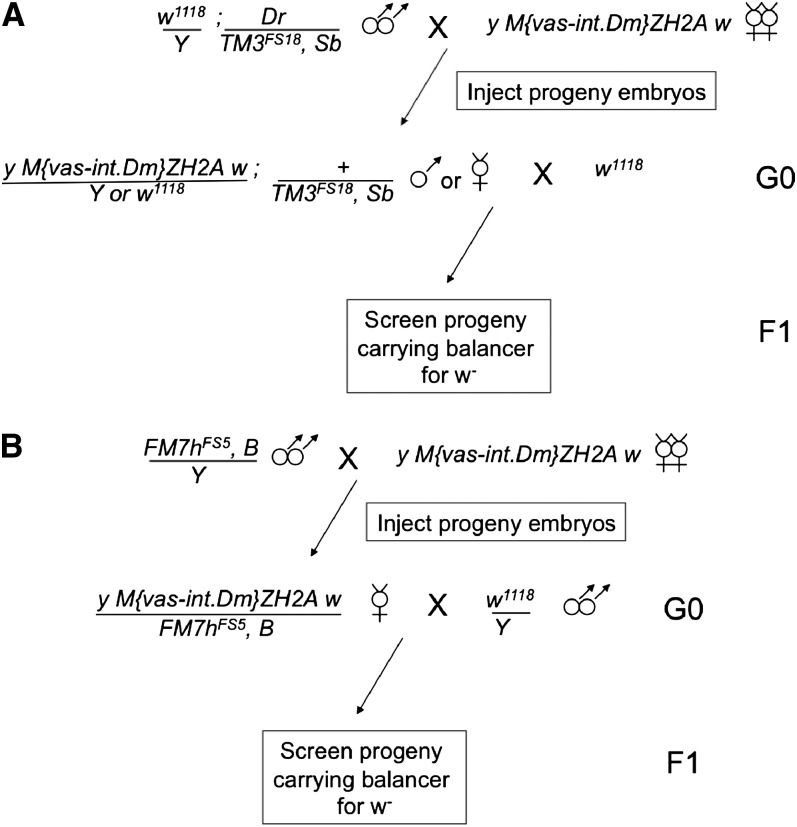
Injection scheme for RMCE using targets on (A) autosomal or (B) X chromosomal balancers. Germline-targeted phiC31 integrase is supplied from the X-chromosomal ZH2A insertion (Bischof *et al.* 2007). In the G0 generation, single males or females (A) or females only (B) that carry the RMCE cassette-bearing balancer (50% of progeny expected) are mated singly to flies with a *w^−^* genotype, and the F1 generation is screened for balanced progeny in which the *mini-white* eye color of the target cassette is lost. Insertions onto *CyO* are obtained through a scheme analogous to (A). See Figure S1 for an alternate strategy using stocks carrying the integrase source and the target balancer concurrently.

**Table 2:** Target cassettes on balancer chromosomes support RMCE

**Balancer Line**	**Donor Cassette Gene**	**Embryos Injected**	**Vials with RMCE**	**% Vials with RMCE**
*FM7h^FS5^*	*yellow*	175	3/12	25%
*FM7h^FS5^*	*GMR-GFP*	200	1/17	5.9%
*TM3^FS18^*	*yellow*	165	2/13	15%
*TM3^FS18^*	*GMR-GFP*	243	7/17	41%
*CyO^J01^*	*yellow*	150	4/26	15.4%
*CyO^J01^*	*GMR-GFP*	150	2/28	7.1%
*CyO^J08^*	*GMR-mCherry*	250	2/19	10.5%

Donor constructs in sterile water were injected according to the scheme in Figure 1. DNA concentrations were 250 ng/µl (*yellow*), 325 ng/µl (*GMR-GFP*), and 115 ng/µl (*GMR-mCherry*).

Finally, we verified that transgene expression was supported in the transformants that we generated. First, we assessed adult body pigmentation of transformants carrying an insertion of the intronless *yellow* gene in an otherwise *yellow* mutant background, and we found that all (9/9) transformed lines produced fully penetrant levels of *yellow* pigmentation indistinguishable from wild-type flies (Figure 2 and data not shown). To address gene expression at earlier points of development, we assessed the expression of donor cassettes carrying *GMR-GFP* or *GMR-mcherry* in whole-mounted late-stage embryos and in eye imaginal discs from wandering third instar larvae (Moses and Rubin 1991). Insertions into *FM7h^FS5^*, *CyO^J01^*, *CyO^J08^*, and *TM3^FS18^* produced robust tissue-specific fluorescence (Figure 2, Figure S2), demonstrating that our modified balancers can support gene expression at multiple stages of development.

**Figure 2:**
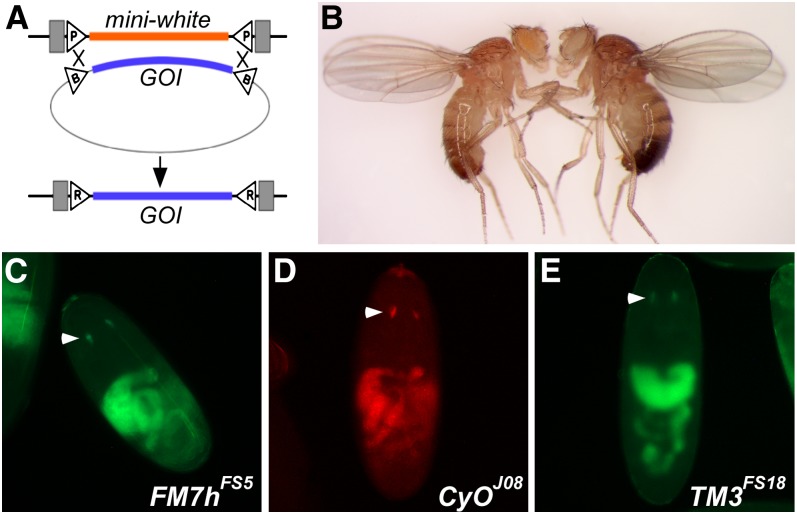
Insertion of donor cassettes onto balancers supports expression of transgenes. (A) Schematic of the exchange reaction. Integrase-mediated crossovers at both ends of the aligned target and donor cassettes result in removal of *mini-white* and integration of the gene of interest (GOI) into the genome. Triangles, att sites; gray boxes, *P*-element ends. (B) *FM7h^FS5^/Y* males before (left) and after (right) RMCE integration of an intronless *yellow* transgene. The fly on the left retains the *mini-white* eye pigmentation and lacks yellow pigmentation, whereas on the right, the *mini-white* eye color is lost, and expression of the *yellow* transgene is evident in the wing and abdomen. Transgenic insertions of the intronless *yellow* cassette on *CyO^J01^* and *TM3^FS18^* produced similar pigmentation (not shown). (C–E) Embryonic expression of *GMR-GFP* (A, C) or *GMR-mCherry* (B) insertions onto balancers. White arrowheads, *GMR*-specific expression (Moses and Rubin 1991); autofluorescence of the gut is also evident.

We anticipate that the modified balancer chromosomes described here will greatly facilitate future efforts at incorporating new transgenes onto balancers and may foster new approaches to balancer chromosome modifications by removing a significant barrier to obtaining balancer insertions. As balancers are developed for other genetic models (Hentges and Justice 2004), a similar scheme for simplified balancer marking may also be beneficial in those systems.

## Supplementary Material

Supporting Information
