# Supplementary material for: Simplified Insertion of Transgenes Onto Balancer Chromosomes via Recombinase-Mediated Cassette Exchange
Source: G3 (Bethesda). 2012 May 1;2(5):551–3. doi: 10.1534/g3.112.002097 (PMC3362938; doi:10.1534/g3.112.002097)
Supplement: Supporting Information [file supp_2_5_551__index.html]

Supporting Information 

# Simplified Insertion of Transgenes Onto Balancer Chromosomes via Recombinase-Mediated Cassette Exchange

## Supporting Information for Sun *et al*, 2012

**Files in this Data Supplement:**

- Supporting Information - Figures S1 and S2, File S1, and Table S1 (PDF, 1.7 MB)
- Figure S1 - Alternate injection scheme for RMCE using a target on a balancer chromosome (PDF, 122 KB)
- Figure S2 - Larval expression of fluorescent reporters inserted onto balancer chromosomes (PDF, 1.6 MB)
- File S1 - Supporting Methods (PDF, 80 KB)
- Table S1 - Local chromosomal features of mapped transgenic insertions (PDF, 76 KB)
