# Supplementary material for: Simplified Insertion of Transgenes Onto Balancer Chromosomes via Recombinase-Mediated Cassette Exchange
Source: G3 (Bethesda). 2012 May 1;2(5):551–3. doi: 10.1534/g3.112.002097 (PMC3362938; doi:10.1534/g3.112.002097)
Supplement: Supporting Information [file supp_2.5.551_002097SI.pdf]

File S1

Supporting Methods

Obtaining insertions of P{attP.w+.attP} onto balancer chromosomes

We used slightly different approaches to obtain insertions onto the autosomal balancers *TM3* and *CyO* as versus the X chromosome balancer *FM7h*. To obtain insertions onto *TM3*, we crossed *y w P{attP.w+.attP}12C; Dr/TM3* females to *y w/Y; Ki (Δ2-3)99B* males, and subsequently mated *y w P{attP.w+.attP}12C/Y; Ki (Δ2-3)99B/TM3* male progeny to w^{1118} females. In the F2, we selected male progeny with *mini-white* eye color, which we assumed resulted from a new insertion of *P{attP.w+.attP}*, and *Sb* bristles, indicating the presence of the *TM3* balancer; of 9052 total F2 flies, we found 156 males with the desired phenotypes. After backcrossing 56 of these males singly to w^{1118} females, we found 26 crosses where the *mini-white* eye color co-segregated with *Sb*, indicating insertions onto the TM3 balancer. We used males from three of these crosses to establish stocks for $TM3^{FS10}$, $TM3^{FS11}$, and $TM3^{FS18}$. Insertions onto *CyO* followed a similar scheme to that used for TM3, but began with an autosomal insertion of *P{attP.w+.attP}*. To obtain insertions onto the X chromosome balancer *FM7h*, we generated females with the genotype *y w P{attP.w+.attP}12C/FM7h; +/TMS (Δ2-3)* and crossed them to w^{1118} */Y* males. Among their progeny, we screened for males with *Bar* eyes, indicating the presence of the *FM7h* balancer, and *mini-white* eye color, indicating a new insertion of the *P{attP.w+.attP}* target cassette. We found 19 w^{+} *Bar* males among 3260 progeny, each of which was backcrossed singly to *C1DX, y f/Y* females. In four crosses, the mini-white phenotype co-segregated with the balancer, from which we established three stocks for $FM7h^{FS2}$, $FM7h^{FS4}$, and $FM7h^{FS5}$. Note that in both cases described here, the number of new insertions onto the balancer relative to elsewhere in the genome was consistent with predictions based on the size of the balancer relative to that of possible targets; in the case of *TM3*, roughly half of new inserts were on the balancer (with possible targets of *Y*, *II*, *TM3*, and *IV*), while in the *FM7h* scheme, roughly one fifth of new inserts were on the balancer (with possible targets of *FM7h*, *II*, *III*, and *IV*).

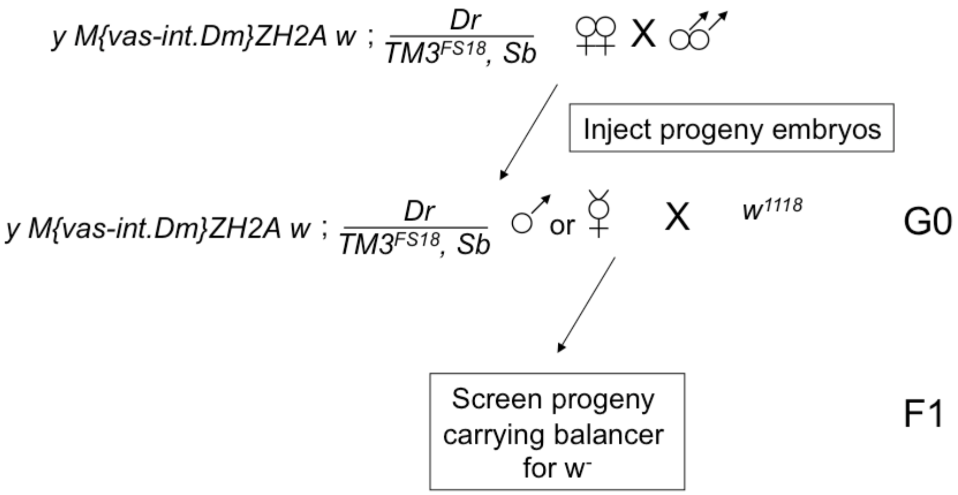

Figure S1 Alternate injection scheme for RMCE using a target on a balancer chromosome. In this scheme, the integrase source and balancer are combined in the stock *y M{vas-int.Dm}ZH2A w*; *Dr/TM3*^{FS18}, alleviating the need to obtain virgin females. In the G0 generation, 50% of embryos will have the desired genotype on the third chromosome (*Dr/TM3*), while 50% will have a lethal genotype (*Dr/Dr* or *TM3/TM3*); either males or females are mated singly to *w*^{1118}. In the F1, all *w*$^{-}$ *Sb* progeny will represent insertions onto the balancer (males only shown for simplicity, females can also be screened).

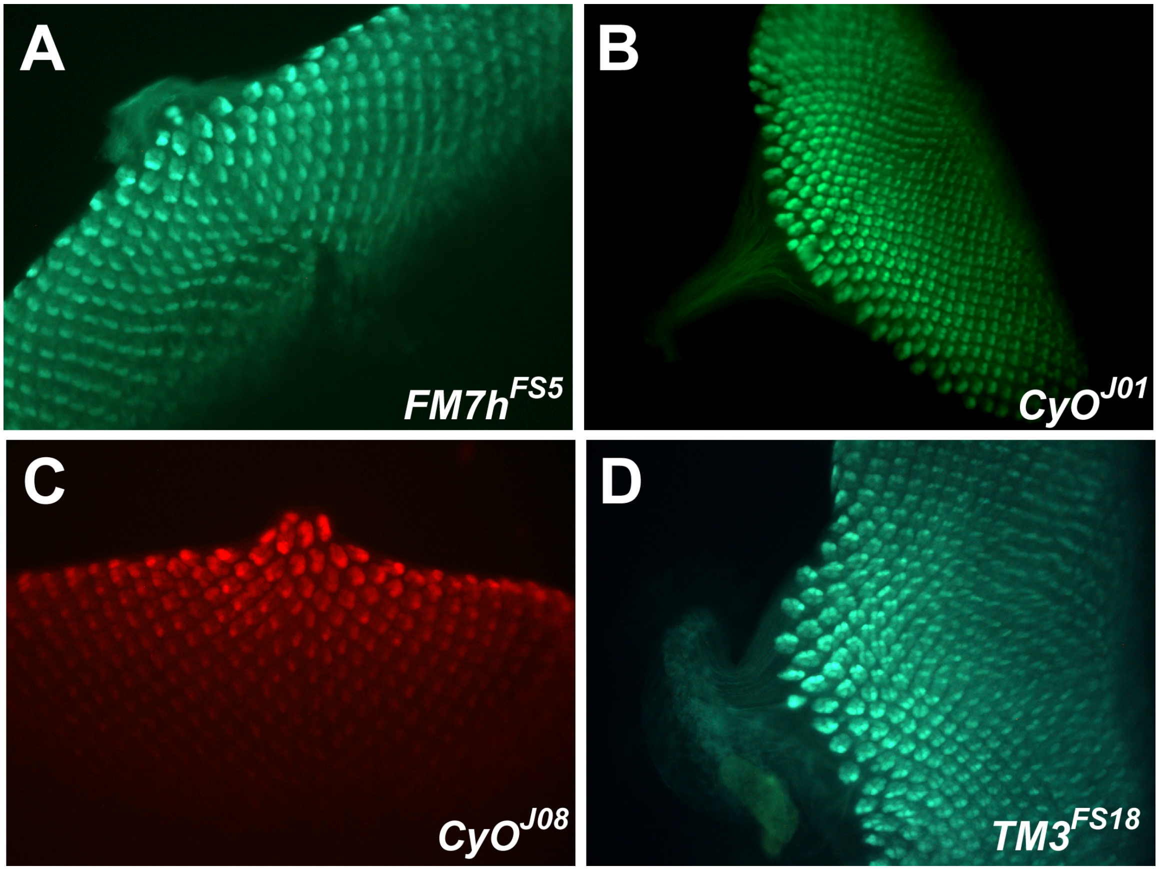

Figure S2 Larval expression of fluorescent reporters inserted onto balancer chromosomes. Eye discs from wandering third instar larvae show expression of *GMR-GFP* (A, B and D) or *GMR-mCherry* (C) inserted onto *FM7h*^{FS5} (A), *CyO*^{J01} (B), *CyO*^{J08} (C), or *TM3*^{FS18} (D).

Table S1 Local chromosomal features of mapped transgenic insertions.

Insertion (Balancer)	Chromatin State[1]		Position Relative to Nearest Gene	RNA-seq Coverage of Developmental Stages[2] for Nearest Gene
	Kc	BG3 S2		
J04 (CyO)	Active euchromatin, usu. broadly expressed	Active promoter/ transcription start	20 nt into first exon of *CG10641*	*CG10641* is expressed throughout development
J08 (CyO)	Active euchromatin, usu. broadly expressed	Active transcribed intron (enhancer)	First intron of *spi*	*spi* is expressed throughout development
FSX4 (FM7h)	Silent, but probably dynamic	Actively transcribed exon on male X	Exon of C, intron of F and B transcripts of *RhoGAP*	*RhoGAP* is expressed throughout development
FSX5 (FM7h)	Silent, but probably dynamic	Actively transcribed exon on male X	400 nt downstream of *Sxl*	*Sxl* is expressed throughout development
FSX2 (FM7h)	Active euchromatin, usu. dev. regulated	Actively transcribed exon on male X	First intron of *dlg1*	*dlg1* is expressed throughout development
FSIII18 (TM3)	Active euchromatin, usu. dev. regulated	Actively transcribed intron	First intron of *ttk*	*ttk* is expressed throughout development
FSIII10 (TM3)	Active euchromatin, usu. dev. regulated	Active promoter/ transcription start region	Second intron of *mub*	*mub* is expressed throughout development
FSIII11 (TM3)	Active euchromatin, usu. broadly expressed	Active promoter/ transcription start region	1 nt into first exon of *CG8043*	*CG8043* is expressed throughout development

[1](Filion *et al.* 2010; Kharchenko *et al.* 2011)
2(Graveley *et al.* 2011)

Supporting References

FILION, G. J., J. G. VAN BEMMEL, U. BRAUNSCHWEIG, W. TALHOUT, J. KIND *et al.*, 2010 Systematic protein location mapping reveals five principal chromatin types in Drosophila cells. Cell **143:** 212-224.

GRAVELEY, B. R., A. N. BROOKS, J. W. CARLSON, M. O. DUFF, J. M. LANDOLIN *et al.*, 2011 The developmental transcriptome of Drosophila melanogaster. Nature **471:** 473-479.

KHARCHENKO, P. V., A. A. ALEKSEYENKO, Y. B. SCHWARTZ, A. MINODA, N. C. RIDDLE *et al.*, 2011 Comprehensive analysis of the chromatin landscape in Drosophila melanogaster. Nature **471:** 480-485.
