# Supplementary material for: Simplified Insertion of Transgenes Onto Balancer Chromosomes via Recombinase-Mediated Cassette Exchange
Source: G3 (Bethesda). 2012 May 1;2(5):551–3. doi: 10.1534/g3.112.002097 (PMC3362938; doi:10.1534/g3.112.002097)
Supplement: Supporting Information [file supp_2.5.551_FigureS1.pdf]

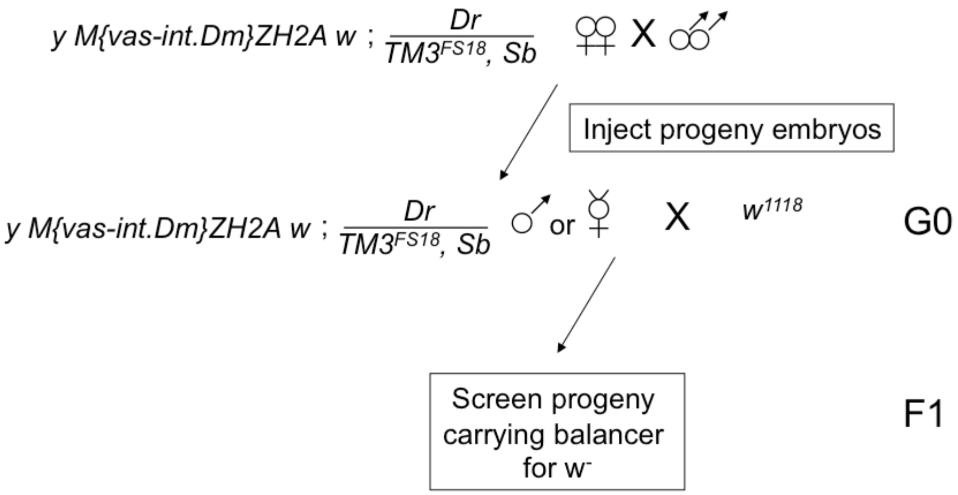

Figure S1 Alternate injection scheme for RMCE using a target on a balancer chromosome. In this scheme, the integrase source and balancer are combined in the stock *y M{vas-int.Dm}ZH2A w*; *Dr/TM3*^{FS18}, alleviating the need to obtain virgin females. In the G0 generation, 50% of embryos will have the desired genotype on the third chromosome (*Dr/TM3*), while 50% will have a lethal genotype (*Dr/Dr* or *TM3/TM3*); either males or females are mated singly to *w*^{1118}. In the F1, all *w*$^{-}$ *Sb* progeny will represent insertions onto the balancer (males only shown for simplicity, females can also be screened).
