# Supplementary material for: Simplified Insertion of Transgenes Onto Balancer Chromosomes via Recombinase-Mediated Cassette Exchange
Source: G3 (Bethesda). 2012 May 1;2(5):551–3. doi: 10.1534/g3.112.002097 (PMC3362938; doi:10.1534/g3.112.002097)
Supplement: Supporting Information [file supp_2.5.551_FigureS2.pdf]

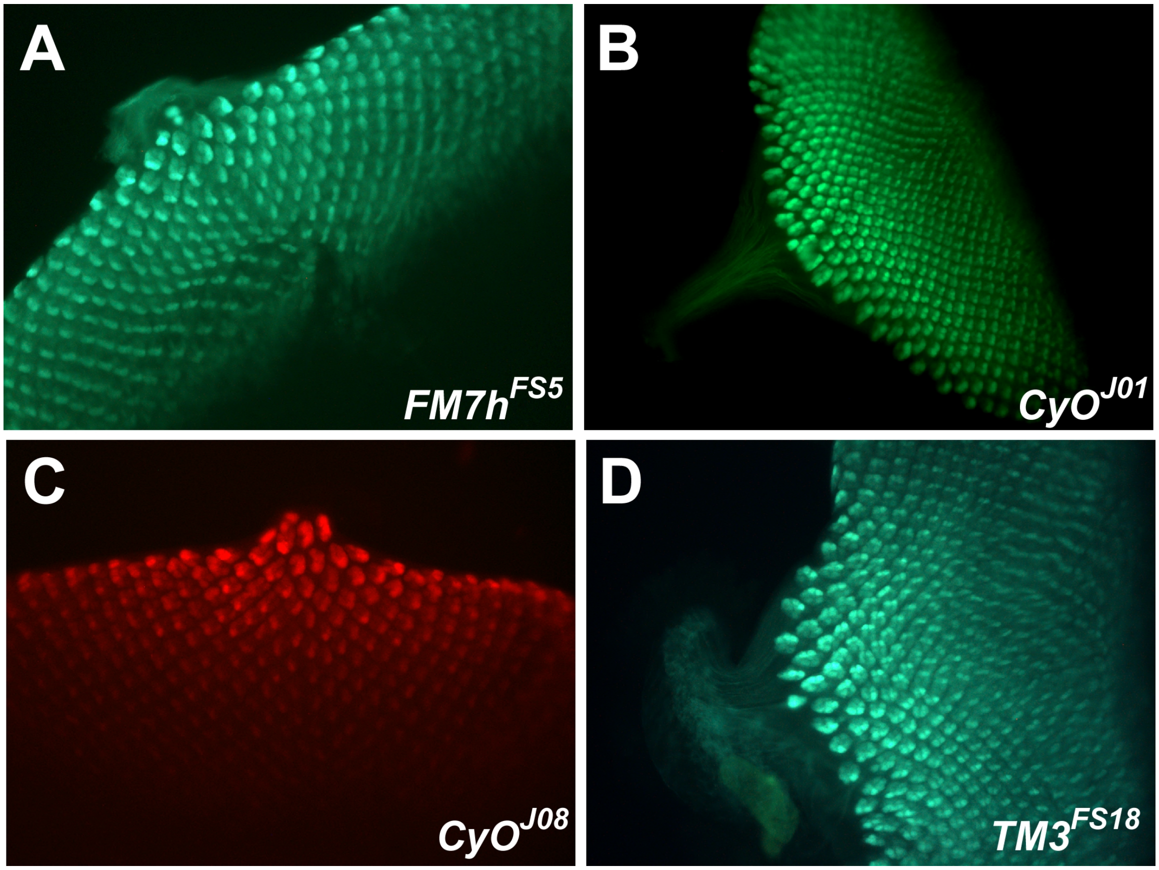

Figure S2 Larval expression of fluorescent reporters inserted onto balancer chromosomes. Eye discs from wandering third instar larvae show expression of *GMR-GFP* (A, B and D) or *GMR-mCherry* (C) inserted onto *FM7h*^{FS5} (A), *CyO*^{J01} (B), *CyO*^{J08} (C), or *TM3*^{FS18} (D).
