# Supplementary material for: Simplified Insertion of Transgenes Onto Balancer Chromosomes via Recombinase-Mediated Cassette Exchange
Source: G3 (Bethesda). 2012 May 1;2(5):551–3. doi: 10.1534/g3.112.002097 (PMC3362938; doi:10.1534/g3.112.002097)
Supplement: Supporting Information [file supp_2.5.551_FileS1.pdf]

File S1

Supporting Methods

Obtaining insertions of P{attP.w+.attP} onto balancer chromosomes

We used slightly different approaches to obtain insertions onto the autosomal balancers *TM3* and *CyO* as versus the X chromosome balancer *FM7h*. To obtain insertions onto *TM3*, we crossed *y w P{attP.w+.attP}12C; Dr/TM3* females to *y w/Y; Ki (Δ2-3)99B* males, and subsequently mated *y w P{attP.w+.attP}12C/Y; Ki (Δ2-3)99B/TM3* male progeny to w^{1118} females. In the F2, we selected male progeny with *mini-white* eye color, which we assumed resulted from a new insertion of *P{attP.w+.attP}*, and *Sb* bristles, indicating the presence of the *TM3* balancer; of 9052 total F2 flies, we found 156 males with the desired phenotypes. After backcrossing 56 of these males singly to w^{1118} females, we found 26 crosses where the *mini-white* eye color co-segregated with *Sb*, indicating insertions onto the TM3 balancer. We used males from three of these crosses to establish stocks for $TM3^{FS10}$, $TM3^{FS11}$, and $TM3^{FS18}$. Insertions onto *CyO* followed a similar scheme to that used for TM3, but began with an autosomal insertion of *P{attP.w+.attP}*. To obtain insertions onto the X chromosome balancer *FM7h*, we generated females with the genotype *y w P{attP.w+.attP}12C/FM7h; +/TMS (Δ2-3)* and crossed them to w^{1118} */Y* males. Among their progeny, we screened for males with *Bar* eyes, indicating the presence of the *FM7h* balancer, and *mini-white* eye color, indicating a new insertion of the *P{attP.w+.attP}* target cassette. We found 19 w^{+} *Bar* males among 3260 progeny, each of which was backcrossed singly to *C1DX, y f/Y* females. In four crosses, the mini-white phenotype co-segregated with the balancer, from which we established three stocks for $FM7h^{FS2}$, $FM7h^{FS4}$, and $FM7h^{FS5}$. Note that in both cases described here, the number of new insertions onto the balancer relative to elsewhere in the genome was consistent with predictions based on the size of the balancer relative to that of possible targets; in the case of *TM3*, roughly half of new inserts were on the balancer (with possible targets of *Y*, *II*, *TM3*, and *IV*), while in the *FM7h* scheme, roughly one fifth of new inserts were on the balancer (with possible targets of *FM7h*, *II*, *III*, and *IV*).
