# Supplementary material for: Simplified Insertion of Transgenes Onto Balancer Chromosomes via Recombinase-Mediated Cassette Exchange
Source: G3 (Bethesda). 2012 May 1;2(5):551–3. doi: 10.1534/g3.112.002097 (PMC3362938; doi:10.1534/g3.112.002097)
Supplement: Supporting Information [file supp_2.5.551_TableS1.pdf]

Table S1 Local chromosomal features of mapped transgenic insertions.

Insertion (Balancer)	Chromatin State[1]		Position Relative to Nearest Gene	RNA-seq Coverage of Developmental Stages[2] for Nearest Gene
	Kc	BG3 S2		
J04 (CyO)	Active euchromatin, usu. broadly expressed	Active promoter/ transcription start	20 nt into first exon of *CG10641*	*CG10641* is expressed throughout development
J08 (CyO)	Active euchromatin, usu. broadly expressed	Active transcribed intron (enhancer)	First intron of *spi*	*spi* is expressed throughout development
FSX4 (FM7h)	Silent, but probably dynamic	Actively transcribed exon on male X	Exon of C, intron of F and B transcripts of *RhoGAP*	*RhoGAP* is expressed throughout development
FSX5 (FM7h)	Silent, but probably dynamic	Actively transcribed exon on male X	400 nt downstream of *Sxl*	*Sxl* is expressed throughout development
FSX2 (FM7h)	Active euchromatin, usu. dev. regulated	Actively transcribed exon on male X	First intron of *dlg1*	*dlg1* is expressed throughout development
FSIII18 (TM3)	Active euchromatin, usu. dev. regulated	Actively transcribed intron	First intron of *ttk*	*ttk* is expressed throughout development
FSIII10 (TM3)	Active euchromatin, usu. dev. regulated	Active promoter/ transcription start region	Second intron of *mub*	*mub* is expressed throughout development
FSIII11 (TM3)	Active euchromatin, usu. broadly expressed	Active promoter/ transcription start region	1 nt into first exon of *CG8043*	*CG8043* is expressed throughout development

[1](Filion *et al.* 2010; Kharchenko *et al.* 2011)
2(Graveley *et al.* 2011)
